# Widely targeted metabolome profiling of different plateau raspberries and berry parts provides innovative insight into their antioxidant activities

**DOI:** 10.3389/fpls.2023.1143439

**Published:** 2023-03-13

**Authors:** Xiaoli Ren, Shulin Wang, Jinying Wang, Dan Xu, Ying Ye, Yangbo Song

**Affiliations:** ^1^ Agriculture and Animal Husbandry College, Qinghai University, Xining, China; ^2^ Department of Public Health, Medical College, Qinghai University, Xining, China

**Keywords:** red raspberry, widely targeted metabolomics, antioxidant activity, antioxidation-related metabolites, correlation analysis

## Abstract

Raspberries are highly nutritious and have powerful antioxidant properties, making them functional berries with positive effects on physiological functioning. However, there is limited information available on the diversity and variability of metabolites in raspberry and its parts, especially in plateau raspberries. To address this, commercial raspberries and their pulp and seeds from two plateaus in China were subjected to LC-MS/MS-based metabolomics analysis and evaluated for antioxidant activity using four assays. A metabolite-metabolite correlation network was established based on antioxidant activity and correlation analysis. The results showed that 1661 metabolites were identified and classified into 12 categories, with significant variations in composition between the whole berry and its parts from different plateaus. Flavonoids, amino acids and their derivatives, and phenolic acids were found to be up-regulated in Qinghai’s raspberry compared to Yunnan’s raspberry. The main differently regulated pathways were related to flavonoid, amino acid, and anthocyanin biosynthesis. The antioxidant activity of Qinghai’s raspberry was stronger than Yunnan’s raspberry, and the order of antioxidant capacity was seed > pulp > berry. The highest FRAP (420.31 µM TE/g DW) values was found in the seed of Qinghai’s raspberry. Overall, these findings suggest that the environment in which the berries grow can affect their chemical composition, and comprehensive exploitation and cultivation of whole raspberry and its parts from different plateaus can lead to new opportunities for phytochemical compositions and antioxidant activity.

## 1 Introduction

Berries, including raspberries, are an excellent source of natural antioxidants and are an essential part of a healthy diet. Red raspberry (*Rubus ideaus* L.) is an aggregate fruit and is a genus of suspension berries (*Rubus*) in the Rosaceae family. Different cultivars and varieties of raspberries are grown worldwide, primarily in Europe, North America, China, Russia, and Japan (Sójka et al., 2016). It is grown for the fresh fruit market and primarily commercial processing into individually quick frozen fruit, juice (Jiang et al., 2022), pulp (Stüwe et al., 2022), dried fruit (Noratto et al., 2017), wine (Cao et al., 2022), and other products. Raspberries have been widely studied by the pharmaceutical, cosmetical, agricultural, and food industries (Kausar and Akhtar, 2016; Gomes et al., 2017; Kirina et al., 2020). Many *in vitro* and *in vivo* investigations on human health have demonstrated that raspberry has antibacterial (Goodman et al., 2020), anti-inflammatory, antioxidant (Wang et al., 2019), antiaging (Kobori et al., 2021), and anticancer (Seeram et al., 2006; Bader Ul Ain et al., 2022). Raspberries are known for their various biological activities, which can be primarily attributed to the presence of a diverse range of phytochemicals, such as flavonoids, phenolic acids, tannins, water-soluble vitamins, amino acids, and lignans (Seeram et al., 2006; Nile and Park, 2014; Huang et al., 2022).

Many studies analyzed the bioactivity and antioxidant capacity of different parts of plants. Afonso et al. (2020) studied bioactive substances and antioxidants in sweet cherry fruit stems and seed kernels. Lenucci et al. (2022) focused on the characterization of several bioactive molecule classes simultaneously performed in different fruit fractions of two mango cultivars. Xi et al. (2017) examined five fruit segments for phenolic chemicals and antioxidant capacity. Nevertheless, prior studies on raspberries has often focused on the whole berry or on specific tissues such as the pulp or seed. Additionally, some studies have only investigated a limited number of metabolites, rather than looking at the global profile of compounds present in raspberries and its parts. A whole raspberry comprises around 100 drupelets, each with a juicy pulp and a single central seed (Iannetta et al., 2000). Usually, red raspberries are eaten in whole fresh berries, and different parts of raspberry, such as pulp and seed, show various phytochemicals composition with distinctive antioxidant activities. Anthocyanins, flavanols, vitamins, superoxide dismutase, and phenolic acids are present in raspberry pulp, which may be advantageous to health (Badin et al., 2023), which are associated with different organoleptic attributes of color, aroma, and taste (Zhao et al., 2022). Although many research articles have been published on the valorization of byproducts from the agroindustry (Marcillo-Parra et al., 2021), the seed of some fruit remains under investigation. Raspberries’ seeds, unlike pulp, contain 10% oil with a unique chemical composition, making them a viable fatty raw material (Kosmala et al., 2015). Raspberry seeds are rich sources of polyunsaturated fatty acids and antioxidants, namely, polyphenols, flavonoids, and ellagitannins, that may improve the antioxidative status of a consumer (Gođevac et al., 2009; Kosmala et al., 2015). As far as we know, oxidative stress leads to various diseases (Gao et al., 2019). The health effects of raspberry, including its seeds, can prevent cancer by inhibiting cell proliferation, inducing autophagy, and inducing apoptosis (Bilawal et al., 2021).

Plant metabolomics can provide a comprehensive analysis of metabolites, a novel technology to analyze the quality and quantity of all metabolites in plants and organs (Li et al., 2021; Segla Koffi Dossou et al., 2022). To date, widely targeted metabolome, based on UPLC-ESI-triple quadrupole-linear ion trap (QTRAP)-MS/MS with a multiple reaction monitoring (MRM) mode, combines the merits of targeted and nontargeted metabolomics. With widely targeted metabolomics analysis, thousands of metabolites can be quickly detected and accurately quantified to assess the metabolome underlying the phenotype of organisms and investigate metabolite variability among different organs, varieties, and species (Scalbert et al., 2011). This approach has been widely used in fruit metabolite analysis, including peach (Gedük and Atsız, 2022), kiwifruit (Li et al., 2023), cherry (Yang et al., 2021), and blueberry (Zheng et al., 2020).

The antioxidant capabilities and bioactive chemicals composition of these berries, pulp, and seeds are not fully understood, despite the nutritional and commercial significance of raspberries. Moreover, studies have shown that raspberry polyphenols have antioxidant properties (Lebedev et al., 2022). Commercial raspberries, their pulp, and seeds from two plateaus (Yunnan and Qinghai, China) were used to confirm the diversity and variability of metabolites in whole raspberries and berry components and to reveal crucial metabolites and pathways causing variations in antioxidant activity. Several studies have documented that the raspberries’ composition and bioactivity are affected by some factors, such as the edaphoclimatic conditions of the growing sites, among others (Kafkas et al., 2008; de Souza et al., 2014; Vara et al., 2020). So, the purpose of this research was to investigate the antioxidant properties of different parts of red raspberries grown in the Qinghai-Xizang Plateau and Yunnan-Guizhou Plateau. We used UPLC-MS/MS-based widely targeted metabolome profiling to compare the metabolites present in the berry, pulp, and seeds of the raspberries and combined these findings with biochemical indicators to identify the key metabolites responsible for the antioxidant properties of red raspberries. This study provides new information on the phytochemical composition of different regions and parts of red raspberries, which could offer valuable insights for the wider cultivation and exploitation of red raspberries.

## 2 Materials and methods

### 2.1 Berry materials and sample preparation

Qinghai raspberries belong to species *Autumn Bliss*, which were harvested at commercial maturity from the red raspberry base of Datong County (36°80’ N, 101°63’ E, altitude: 2280 m), Qinghai, Qinghai-Xizang Plateau. For Yunnan raspberries belong to species *Heritage*, or the classical ‘Driscoll’s’, one of the finest fresh berry producers across the globe, which were collected from an orchard in Zhanyi Area (27°49’ N, 103°80’ E, altitude: 2000 m), Yunnan, Yunnan-Guizhou Plateau. The average annual temperature, annual sunshine hours, and rainfall of Datong County were 4.9°C, 2553 h, and 523 mm, respectively, and those of the Zhanyi Area were 17.4°C, 2098 h, and 1002 mm. The chosen berries were uniformly colored and free of mechanical damage. Fresh berries were transported to the lab in a –18°C cold chamber. For the preparation of pulp, the defrosted berry was extracted on a low-speed juicer (model number JYL-C93T, Joyoung Company Limited, China), and the seeds and pulp were collected separately. According to locations and tissues (berry, pulp, and seeds), the red raspberry’s sample from Qinghai-Xizang Plateau was marked as Q-R, Q-RP, and Q-RS, and the red raspberry’s sample from Yunnan-Guizhou Plateau was noted as Y-R, Y-RP, and Y-RS. Samples were stored at –80°C until analysis.

The samples are freeze-dried with a vacuum freeze-dryer (Scientz-100F). The freeze-dried sample was crushed using a mixer mill (MM 400, Retsch, Germany) with a zirconia bead for 1.5 min at 30 Hz. Dissolve 50 mg of lyophilized powder with 1.2 mL 70% methanol solution, vortex 30 sec per 30 min for 6 rounds. Following centrifugation at 12000 rpm for 3 min, the supernatant was collected and filtered by a microporous membrane filter (0.22 μm pore size) and stored in the sample injection bottle for UPLC-MS/MS analysis.

### 2.2 Chemicals

Methanol, acetonitrile, and ethanol were all chromatographic purity purchased from Merck (Darmstadt, Hesse, Germany). Formic acid was purchased from Aladdin (Shanghai, China). Standard compounds such as gallic acid and (+)-catechin were obtained from Yuanye company (Shanghai, China). The remaining chemical reagents were purchased from Sinopharm Co. Ltd. (Shanghai, China).

### 2.3 Widely-targeted metabolomics analysis

After sample preparation, the sample was performed at Metware (Wuhan, China) for widely-targeted metabolomics measurement. The operation process was strictly following the operation flow of the UPLC and MASS spectrometry. The column, Agilent SB-C18 (1.8 μm, 2.1 mm * 100 mm), was used, and the mobile phase consisted of solvent A (pure water with 0.1% formic acid) and solvent B (acetonitrile with 0.1% formic acid). Samples were measured using a gradient program with 95% A and 5% B as starting conditions. Within 9 min, a linear gradient to 5% A, 95% B was programmed, and a composition of 5% A, 95% B was held for 1 min. Subsequently, a composition of 95% A, 5% B was adjusted within 1.1 min and kept for 2.9 min. The flow rate was 0.35 mL/min, the column temperature was 40°C, and the injection volume was 4 μL. Data filtration, alignment, and calculation were carried out using Analyst 1.6.1 software (AB SCIEX Pet. Ltd, Framingham, Massachusetts, USA). The ESI source operation parameters were as follows: (1) source temperature 550°C; (2) ion spray voltage (IS) 5500 V (positive ion mode)/– 4500 V (negative ion mode); ion source gas I (GSI), gas II(GSII), and curtain gas (CUR) were set at 50, 60, and 25 psi, respectively; the collision-activated dissociation (CAD) was high. Triple quadrupole (QQQ) scans were acquired as multiple reaction monitoring (MRM) experiments with optimized declustering potential (DP) and collision energy (CE) for each individual MRM transition, and a specific set of the transitions were monitored during each period based on the eluted metabolites (Zhu et al., 2013).

### 2.4 Antioxidant activities

#### 2.4.1 DPPH method

For DPPH assay, the procedure followed the method of Afonso et al. (2020) with some modifications. Briefly, 15 mL of the extract and 15 mL of 0.1 mM DPPH solution were added to a test tube and incubated for 30 min in dark. Two hundred microliters of the final mixture were added to a microplate for absorbance determination at 517 nm. Assays were performed after appropriate dilution for samples with high antioxidant activity. 80% methanol was used as control, and Trolox was used as standard. The results were expressed as μg Trolox equivalent per g of fresh weight (μg TE/g FW).

#### 2.4.2 FRAP method

The ferric ion reducing antioxidant power (FRAP) assay was also performed according to the method reported by Li et al. (2012). with some modifications. Briefly, 40 μL of extraction solution diluted with distilled water (1:20, v/v) was mixed with 200 μL of freshly prepared ferric-tripyridyl-triazine agent (Fe^3+^-TPTZ) in a 96-well microplate. Fe^3+^-TPTZ was prepared by mixing 20 mM FeCl_3_·6H_2_O, 10 mM TPTZ in 40 mM HCl, and 300 mM acetate buffer in a 1:1:10 (v/v/v) ratio. The plates were maintained in the dark at 25°C for 30 min, and their absorbances at 593 nm were measured., and the results were expressed as µM Trolox equivalent per g of dry weight (μM TE/g DW) as the mean ± standard deviation (SD) of three replicates.

#### 2.4.3 ABTS method

The ABTS radical scavenging activity was evaluated in a 96-well microplate using the method of Re et al. (1999). An ABTS radical solution was prepared by mixing 7 mM of ABTS at pH 7.4 (5 mM NaH_2_PO_4_, 5 mM Na_2_HPO_4_, and 154 mM NaCl) with 2.5 mM potassium persulfate and storing the mixture in the dark at room temperature for 16 h. The mixture was then diluted with ethanol to give an absorbance of 0.70 ± 0.02 units at 734 nm. In each microplate well, 15 µL of the extract was mixed with 285 µL of the freshly prepared ABTS solution and incubated at room temperature in the dark for 10 min. A standard calibration curve was constructed for Trolox at 0, 80, 160, 240, 320, and 400 μmol/L concentrations. Absorbance values were measured at 734 nm, and the results were expressed as µM Trolox equivalent per g of dry weight (μM TE/g DW) as the mean ± standard deviation (SD) of three replicates.

#### 2.4.4 ORAC

ORAC is based on a hydrogen atom transfer (HAT) process, with the oxidation of a fluorescent probe by peroxyl radicals. The procedure for the ORAC assay was performed on plasma according to the instructions supplied with the Oxygen Radical Antioxidant Capacity (ORAC) assay kit from CELL BIOLABS, INC (San Diego, USA). The kit included a 96-well microtiter plate with clean bottom black plate, fluorescein probe 100 ×, free radical initiator, antioxidant standard (Trolox™), and assay diluent (4 ×). The samples were dissolved in a ratio of 1:100 (Rolnik et al., 2021).

### 2.5 Data analysis

The antioxidant ability’s results were expressed as the mean ± standard deviation (SD) using SPSS 17.0 (Chicago, IL, USA). One-way analysis of variance (ANOVA) and Tukey’s test were applied to establish the significance of the differences among samples (*p* < 0.05). Origin Pro software (2021b, OriginLab Inc.) was used for image processing. The metabolomic data were processed by multivariate statistical analysis methods, including principal component analysis (PCA), hierarchical cluster analysis (HCA), and orthogonal partial least squares discriminant analysis (OPLS-DA). PCA was first performed on all samples (including QC samples) to determine the overall metabolite differences as well as the variation degree among the red raspberries samples. The HCA (Hierarchical Cluster Analysis) results for samples and metabolites are presented as heat maps and are performed by the R package Complex Heatmap. Orthogonal partial least squares-discriminant analysis (OPLS-DA) was generated using the Metabo Analyst R package. Identified metabolites were annotated using Kyoto Encyclopedia of Genes and Genomes (KEGG) compound databases (http://www.kegg.jp/kegg/compound/ ) and Metware database, which were then mapped to KEGG Pathway database (http://www.kegg.jp/kegg/pathway.html ), followed by enrichment and topological analysis of the pathways where differential metabolites were present. Key pathways were further screened based on the number of differential metabolites (Xiao et al., 2021).

## 3 Results

### 3.1 Widely targeted metabolomics analysis

**Figure 1:**
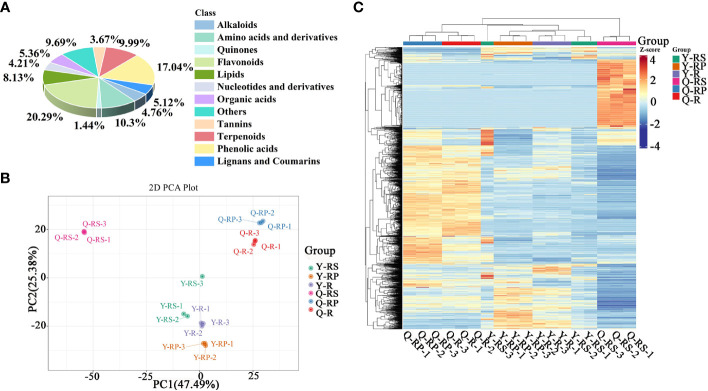
Overview analysis of widely-targeted metabolomics of different plateau raspberries. Classifications of these metabolites identified based on the mass-to-charge ratio of the compounds in Metware database **(A)**; PCA analysis **(B)**; hierarchical cluster analysis **(C)**.

In order to get a complete picture of the differences in the phytochemical composition of red raspberries, widely targeted metabolomics of raspberries and different parts (berry, pulp, and seeds) was performed using UPLC-ESI-MS/MS. Totally 1661 metabolites were identified in **Supplementary Table S1**. These metabolites were classified into 12 categories, among which 283 phenolic acids, 135 lipids, 337 flavonoids, 89 organic acids, 171 amino acids and derivatives, 70 nucleotides and derivatives, 166 terpenoids, 85 lignans and coumarins, 79 alkaloids, 61 tannins, 161 other, and 24 quinones. Other classes mainly contain sugars, vitamins, aldehydes, ketones, lactones, and others. We found the highest relative content of flavonoids and phenolic acids, with 20.29% and 17.04%, respectively (**Figure 1A**). Analyst 1.6.3 processed mass spectrum data. **Supplemental Figures S1**, **S2** showed the mixed sample’s TIC and MRM metabolite detection multi-peak plots.

### 3.2 Metabolite variations between Qinghai raspberry and Yunnan raspberry

**Figure 2:**
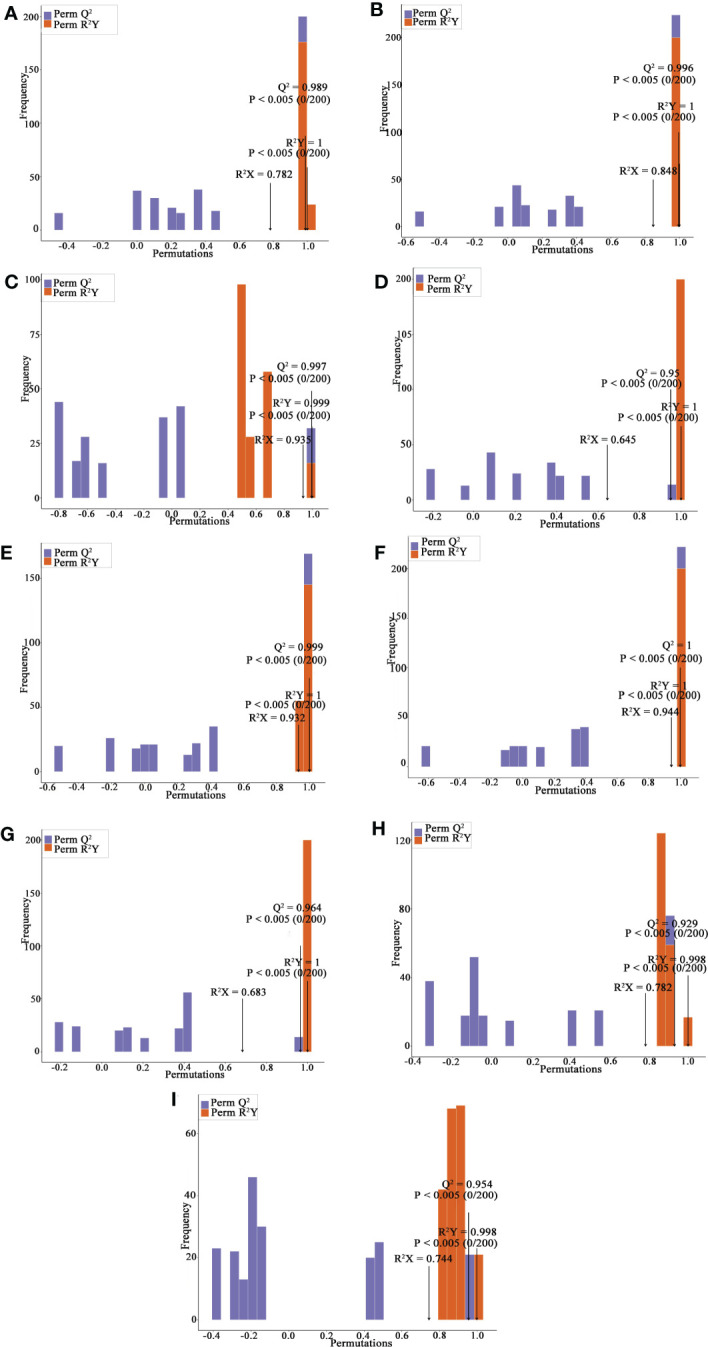
OPLS-DA Validation Chart, Y-R vs Q-R, Y-RP vs Q-RP, Y-RS vs Q-RS, Q-R vs Q-RP, Q-R vs Q-RS, Q-RP vs Q-RS, Y-R vs Y-RP, Y-R vs Y-RS, and Y-RP vs Y-RS **(A-I)**. The horizontal coordinate indicates the model R^2^Y, Q^2^ values, and the vertical coordinate is the frequency of the model classification effect in 200 random permutation experiments.

**Figure 3:**
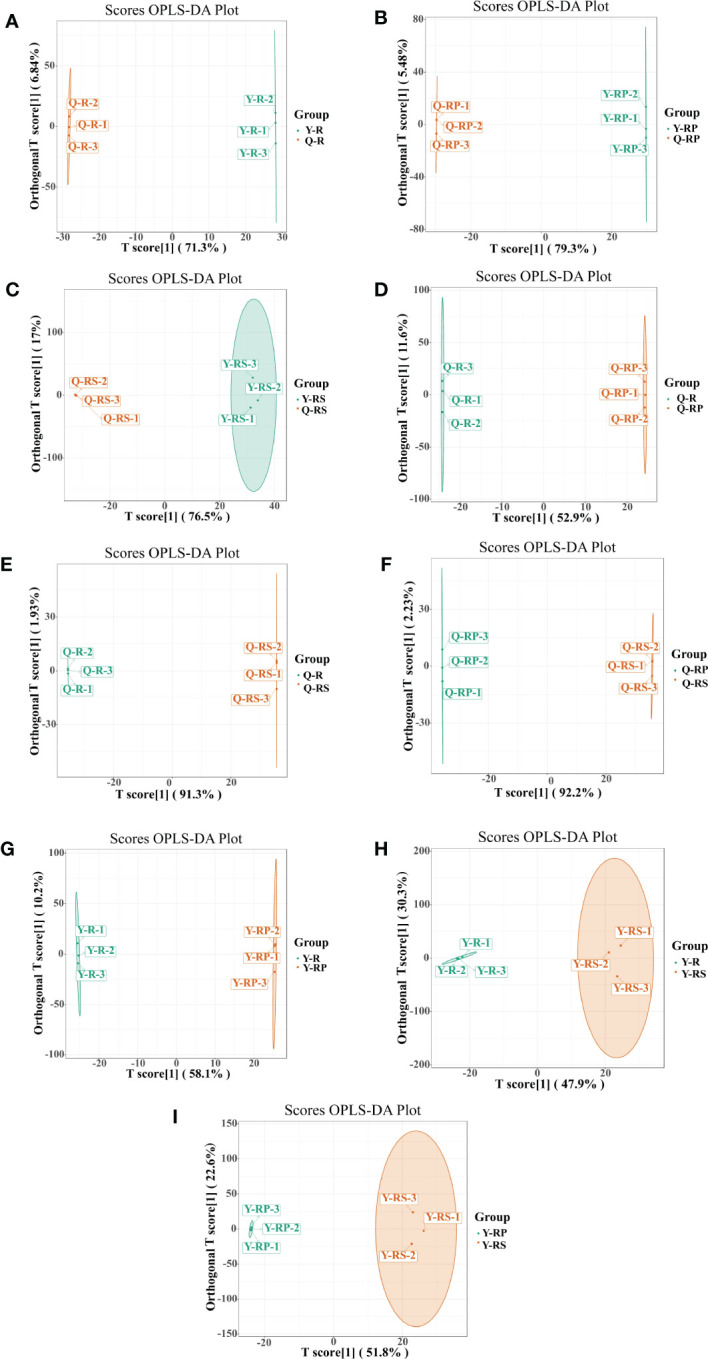
The OPLS-DA score plots of. OPLS-DA model plots for the comparison groups, Y-R vs Q-R, Y-RP vs Q-RP, Y-RS vs Q-RS, Q-R vs Q-RP, Q-R vs Q-RS, Q-RP vs Q-RS, Y-R vs Y-RP, Y-R vs Y-RS, and Y-RP vs Y-RS **(A–I)**.

**Figure 4:**
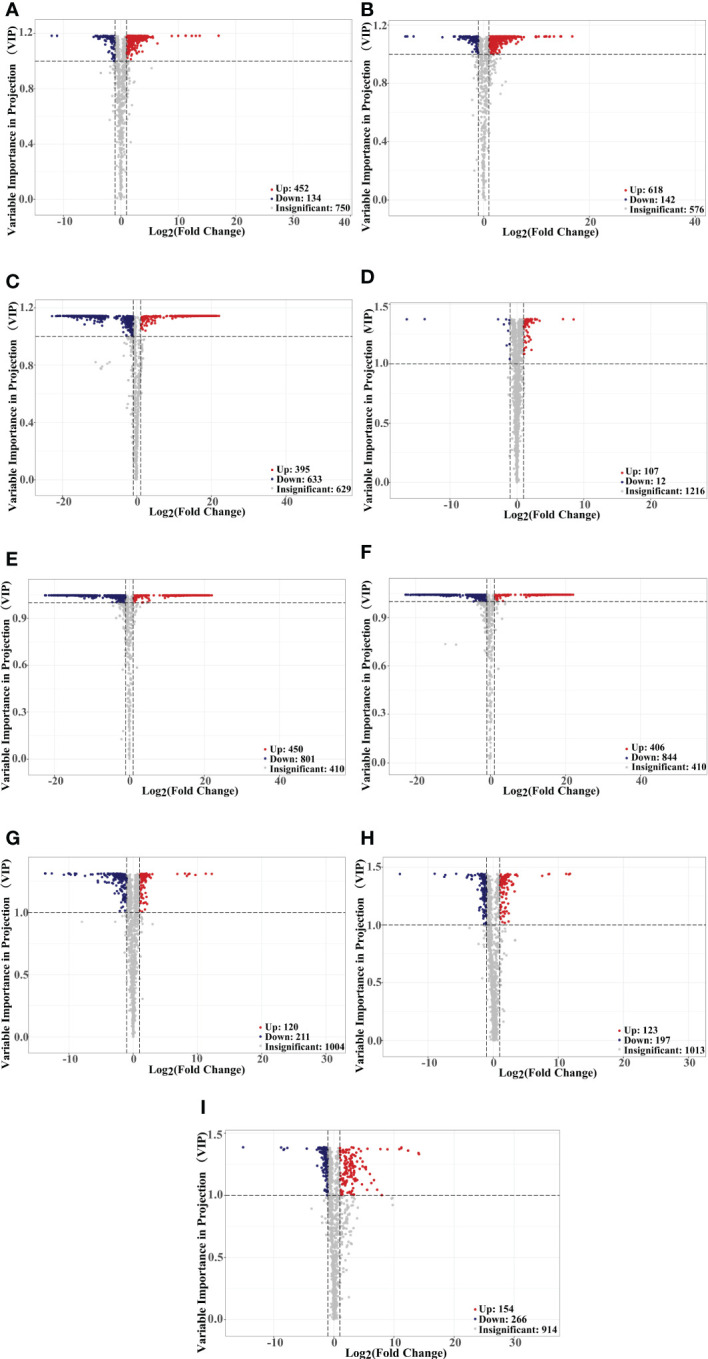
Volcano plots showing the differences in the expression levels of metabolites in red raspberry, red spots indicate up-regulated differentially expressed metabolites; blue spots indicate down-regulated differentially expressed metabolites, and grey spots indicate detected metabolites, the differences were not significant at p < 0.05, Y-R vs Q-R, Y-RP vs Q-RP, Y-RS vs Q-RS, Q-R vs Q-RP, Q-R vs Q-RS, Q-RP vs Q-RS, Y-R vs Y-RP, Y-R vs Y-RS, and Y-RP vs Y-RS **(A–I)**.

Principal component analysis (PCA) is a multivariate method commonly used to summarize data variances, reveal group differences, and measure sample variability within a group (Wang et al., 2018). PC1 contributed 47.49% and PC2 25.38%. Two primary components contributed 72.87% (**Figure 1B**). Separation of samples from other plateau occurred in the second principal component, and PCA classified Qinghai and Yunnan raspberry samples differently. There were significant differences between seed and pulp or berry in Qinghai raspberry metabolites, indicating that the relative quantification of the metabolites was significantly different among different plateaus and parts. The clustering heatmap of the metabolites also clearly showed the similarity between the biological replicates and the differences among the red raspberry from two plateaus (**Figure 1C**). Furthermore, the OPLS-DA model validation Q^2^ indicates the predictive power, R^2^Y and R^2^X indicate the explanation rate of the Y matrix and the X matrix, respectively. And R^2^Y and Q^2^ scores were above 0.9, which means the model was appropriate, *p* < 0.005 model was excellent. We observed high predictability (Q^2^) and strong goodness of fit (R^2^X and R^2^Y). For instance, the Q^2^ values between Y-R vs Q-R, Y-RP vs Q-RP, Y-RS vs Q-RS, Q-R vs Q-RP, Q-R vs Q-RS, Q-RP vs Q-RS, Y-R vs Y-RP, Y-R vs Y-RS, and Y-RP vs Y-RS was 0.989, 0.996, 0.997, 0.95, 0.999, 1, 0.964, 0.929, and 0.954 indicating the metabolite profiles of parts and plateaus were distinctly different (**Figure 2**). OPLS-DA score plots showed that the same parts of red raspberries in different plateaus and different parts of raspberries were well-separated in pairs, suggesting significant differences in metabolic phenotypes of the two kinds of raspberries (**Figure 3**). Respectively, according to VIP ≥ 1 screened from OPLS-DA results and a fold change ≥ 2 or ≤ 0.5. The screening results are presented in volcano plots (**Figure 4**).

**Figure 5:**
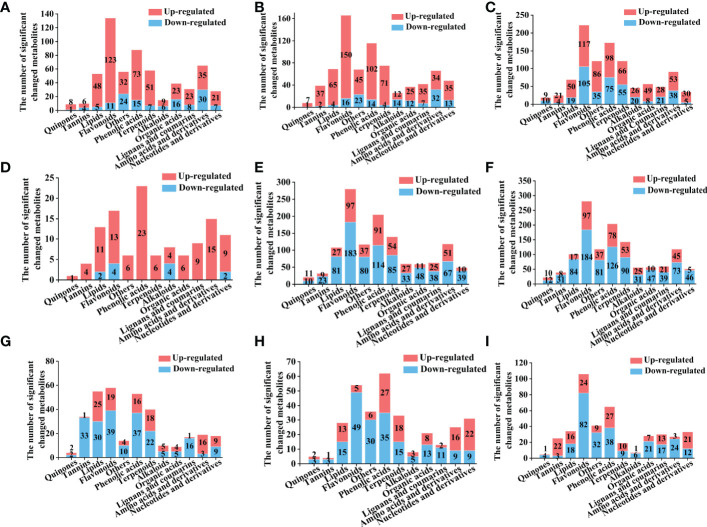
Number of different types of differential metabolites for the comparison group, Y-R vs Q-R, Y-RP vs Q-RP, Y-RS vs Q-RS, Q-R vs Q-RP, Q-R vs Q-RS, Q-RP vs Q-RS, Y-R vs Y-RP, Y-R vs Y-RS, and Y-RP vs Y-RS (A-I). Yellow column indicates metabolites that were significantly up-regulated, blue column indicates metabolites that were significantly down-regulated. Significantly regulated metabolites between groups were determined by VIP ≥ 1 and FC ≥ 2 or ≤ 0.5.

For raspberry from two plateaus, Y-R vs Q-R contained 586 significantly different metabolites (452 up-regulated, 134 down-regulated). Of these, 27.21% of flavonoids, 16.15% of phenolic acids, 11.28% of terpenoids, and 7.74% of amino acids and their derivatives were up-regulated in Q-R. The key differentially regulated metabolites included 49.78% of polyphenols, up-regulated in Qinghai raspberry (**Figures 4A**, **5A**). Y-RP vs Q-RP showed 760 substantially different metabolites (618 up-regulated and 142 down-regulated). Of these, 24.27% of flavonoids, 16.50% of phenolic acids, 11.49% of terpenoids, and 10.52% of lipids were up-regulated in Q-RP (**Figures 4B**, **5B**). Between Y-RS and Q-RS, 1028 substantially different metabolites (395 up-regulated and 633 down-regulated) were found. Among these, the Q-RS showed an up-regulation of 26.58% flavonoids, 18.98% phenolic acids, 13.92% terpenoids, and 9.62% amino acids and their derivatives (**Figures 4C**, **5C**).

The analysis of different parts of raspberry showed significant differences in metabolites between various comparisons. For instance, Q-R vs Q-RP showed 119 differential metabolites, out of which 107 were up-regulated, and 12 were down-regulated. Similarly, Q-R vs Q-RS, Q-RP vs Q-RS, Y-R vs Y-RP, Y-R vs Y-RS, and Y-RP vs Y-RS showed 1251, 1250, 331, 320, and 420 differential metabolites, respectively (**Figures 4D–I**). Flavonoids, phenolic acids, amino acids and derivatives, and terpenoids were the major categories of differential metabolites observed in these comparisons, comprising over 50% of the total differential metabolites (**Figure 5D–I**).

Differential accumulated metabolite (DAM) mainly consisted of phenolic acids, flavonoids, and amino acids and their derivatives, and about half of the DAMs was phenolic acids and flavonoids. These metabolites are important secondary metabolites in many plants. They contribute to the antioxidant activity of plants (Ramalingam et al., 2021). Thus, the difference in DAMs in pairwise comparisons suggests that the functional activity of the raspberries from the two plateaus may differ.

### 3.3 KEGG annotation and enrichment analysis of differential metabolites

**Figure 6:**
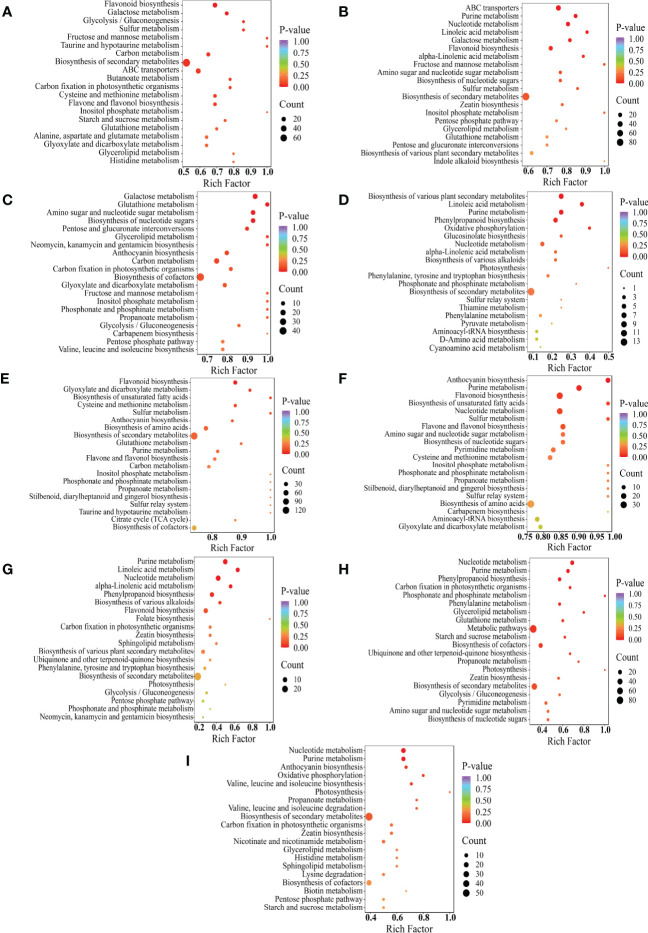
KEGG enrichment of differential metabolites between the comparison groups, Y-R vs Q-R, Y-RP vs Q-RP, Y-RS vs Q-RS, Q-R vs Q-RP, Q-R vs Q-RS, Q-RP vs Q-RS, Y-R vs Y-RP, Y-R vs Y-RS, and Y-RP vs Y-RS **(A–I)**. Each bubble in the plot represents a metabolic pathway whose abscissa and bubble size jointly indicate the magnitude of the impact factors of the pathway. A larger bubble size indicates a larger impact factor. The bubble colors represent the *p*-values of the enrichment analysis, with darker colors showing a higher degree of enrichment. (For interpretation of the references to color in this figure legend, the reader is referred to the web version of this article).

To further conduct the major pathways of DAMs in samples, the KEGG enrichment analysis of each cluster to obtain detailed information about the metabolic pathways was shown in bubble plots (**Figure 6**). The DAMs were enriched into 88, 88, and 97 pathways in Y-R vs Q-R, Y-RP vs Q-RP, and Y-RS vs Q-RS, respectively (**Supplementary Table S2**). Y-R and Q-R occurred in flavonoid biosynthesis, flavone, and flavonol biosynthesis. The primary enrichment of differential metabolites between Y-RP and Q-RP occurred in purine metabolism and flavonoid biosynthesis. In addition, tthe differential metabolites between the Y-RS and Q-RS were involved in anthocyanin biosynthesis, biosynthesis of amino acids. These results indicate that environmental and climate factors have great changes in metabolites in the anthocyanin, flavonoid, flavone, and flavonol biosynthesis pathways.

The DAMs for part’s groups (Q-R vs Q-RP, Q-R vs Q-RS, Q-RP vs Q-RS, Y-R vs Y-RP, Y-R vs Y-RS, and Y-RP vs Y-RS) were involved in 38, 100, 100, 52, 77, and 80 pathways (**Supplementary Table S2**). **Figures 6D-I** displayed the top 20 enriched pathways for the DAMs. Multiple primary metabolism pathways and secondary metabolic pathways (purine metabolism, phenylpropanoid biosynthesis, biosynthesis of various plant secondary metabolites, cysteine and methionine metabolism, flavonoid biosynthesis, anthocyanin biosynthesis, biosynthesis of unsaturated fatty acids, alpha-linolenic acid metabolism, linoleic acid metabolism, valine, leucine, and isoleucine biosynthesis) enriched by these DAMs. In these comparison groups, it is worth noting that these pathways are related to the biosynthesis of flavonoids, phenolic acids, fatty acids, and amino acids and their derivatives. The flavonoid and anthocyanin biosynthesis pathways, for example, are located downstream of the phenylpropanoid biosynthetic pathway (Qiu et al., 2020). In these comparison groups, some metabolic pathways overlapped, such as purine metabolism. The process of purine metabolism serves as a fundamental stage in the synthesis of nucleic acids and is intricately linked to the primary and secondary metabolic pathways of plants, which are also closely associated with the polyphenol metabolic pathway. The metabolic pathways appear to be significantly linked to the potent antioxidant properties observed in raspberry seeds.

### 3.4 Key significantly differential metabolites

**Figure 7:**
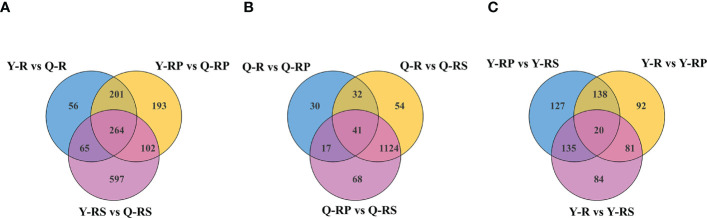
Plotted Venn diagram of the different metabolites in the above comparison groups from raspberries, Y-R vs Q-R, Y-RP vs Q-RP, and Y-RS vs Q-RS Venn diagram, **(A)**; Q-R vs Q-RP, Q-R vs Q-RS, and Q-RP vs Q-RS, **(B)**; Y-R vs Y-RP, Y-R vs Y-RS, and Y-RP vs Y-RS, **(C)**, were plotted using Venn diagram package.

**Figure 8:**
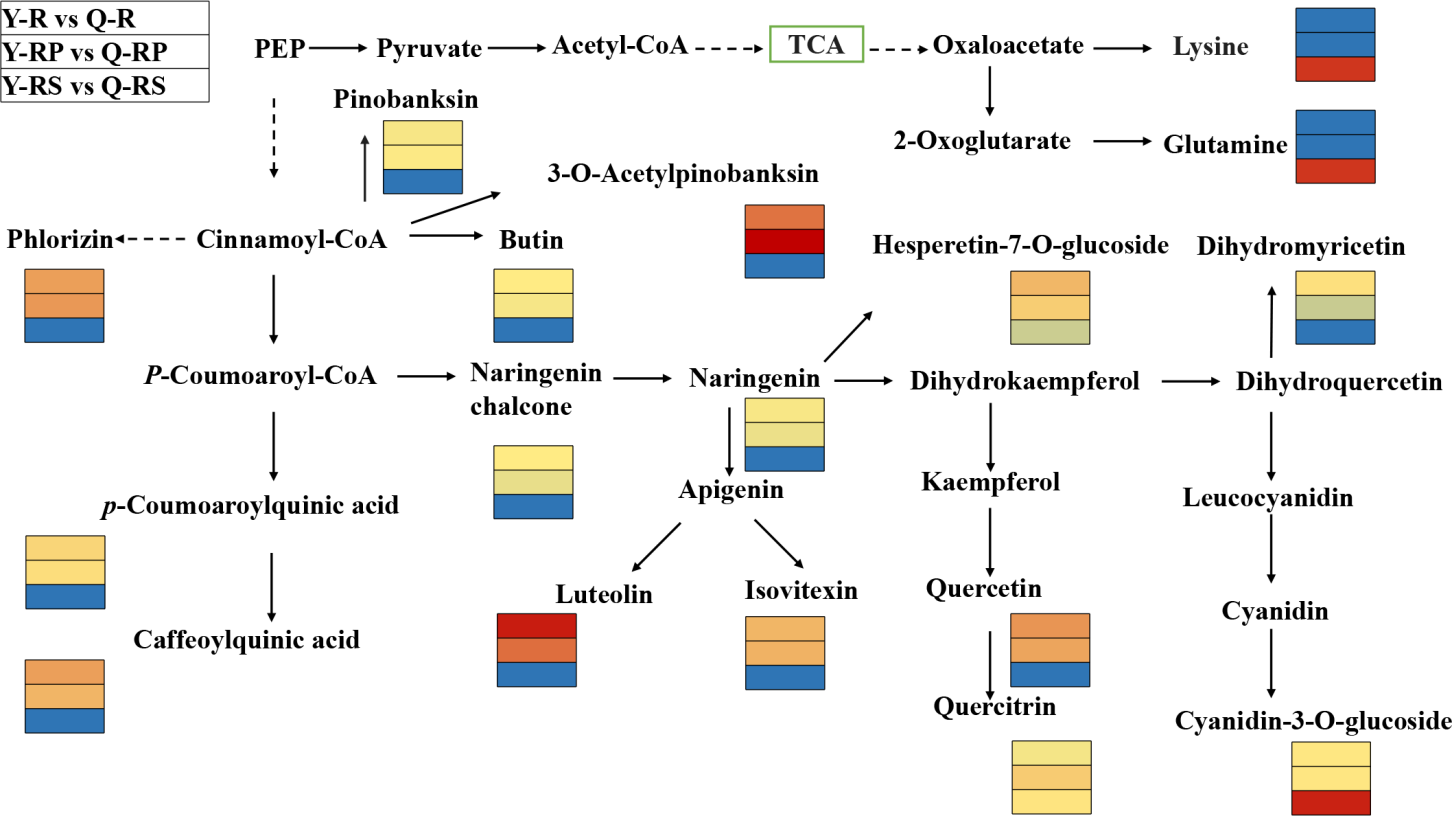
Overview of the probable regulation of some key metabolites mapped to metabolic pathways in pairwise comparisons of the raspberry from different plateaus. The red color small rectangle indicates that the metabolite content is significantly up-regulated; the blue rectangle indicates that the metabolite content is significantly down-regulated.

**Figure 9:**
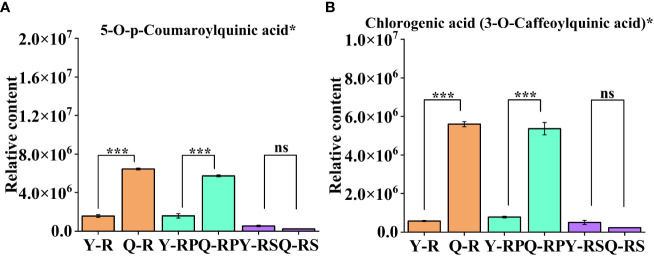
Variation of two selected bioactive phenolic acid in two plateaus. 5-O-p-Coumaroylquinic acid* **(A)**; Chlorogenic acid (3-O-Caffeoylquinic acid)* **(B)**. Bars indicate the s.d. of three replicates. “ns” means not significant, *** p < 0.005.

**Figure 10:**
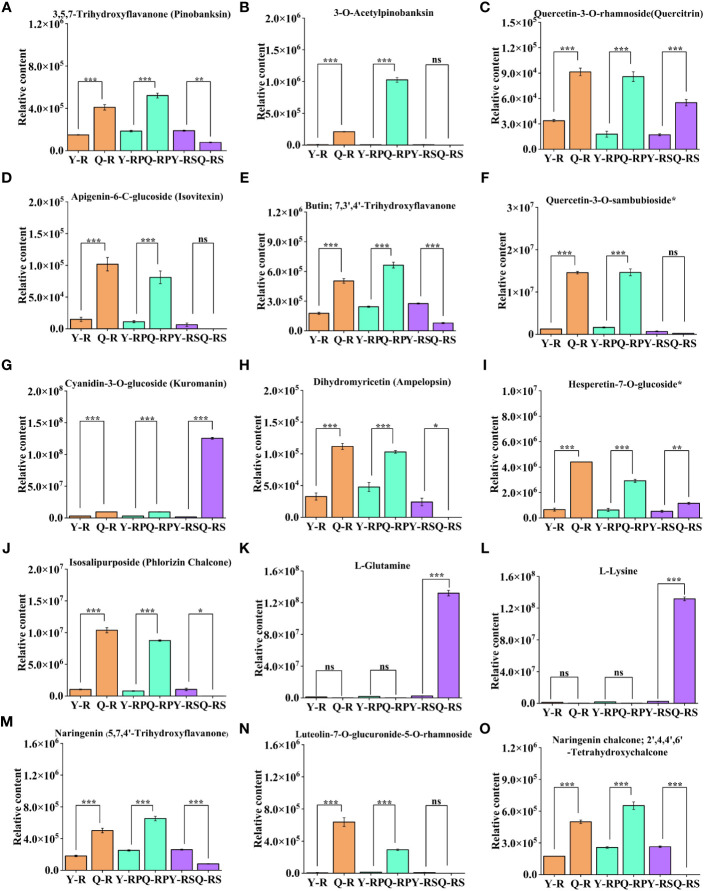
Difference in the relative content of thirteen selected bioactive flavonoids and two amino acids and their derivatives in two plateaus. 3,5,7-Trihydroxyflavanone (Pinobanksin) **(A)**, 3-O-Acetylpinobanksin **(B)**, Quercetin-3-O-rhamnoside(Quercitrin) **(C)**, Apigenin-6-C-glucoside (Isovitexin) **(D)**, Butin; 7,3',4'-Trihydroxyflavanone **(E)**, Quercetin-3-O-sambubioside* **(F)**, Cyanidin-3-O-glucoside (Kuromanin) **(G)**, Dihydromyricetin (Ampelopsin) **(H)**, Hesperetin-7-O-glucoside* **(I)**, Isosalipurposide (Phlorizin Chalcone) **(J)**, L-Glutamine **(K)**, L-Lysine **(L)**, Naringenin (5,7,4'-Trihydroxyflavanone) **(M)**, Luteolin-7-O-glucuronide-5-O-rhamnoside **(N)**, Naringenin chalcone; 2',4,4',6'-Tetrahydroxychalcone **(O)**. Bars indicate the s.d. of three replicates. “ns” means not significant, * *p* < 0.05, ** *p* < 0.01, *** *p* < 0.005.

In order to identify the crucial metabolites present in raspberries grown on various plateaus, a Venn diagram was created to compare the differential metabolites between Y-R and Q-R, Y-RP and Q-RP, and Y-RS and Q-RS (**Figure 7A**). We discovered that the majority of these metabolites, which were among the 264 that overlapped between Y-R and Q-R, Y-RP and Q-RP, and Y-RS and Q-RS, were up-regulated in Qinghai raspberries. (**Supplementary Table S3**). There were 43.56% and 7.58% respectively of polyphenolic compounds and amino acids and their derivatives. Also, the data were merged into the appropriate maps based on the KEGG annotation, and the metabolic pathways of the most pertinent overlapping differential metabolites (**Figure 8**). We took the up-regulated metabolites in metabolic pathways related to antioxidant properties as the standard and screened out three differential metabolites related to antioxidant quality in the overlapping substances. The anthocyanin biosynthesis, flavonoid biosynthesis, flavone and flavonol biosynthesis, and biosynthesis of amino acids were enriched among the different comparison groups. Some metabolites of raspberries grown in QZP, including Chlorogenic acid (3-O-Caffeoylquinic acid)*, 5-O-p-Coumaroylquinic acid*, Luteolin-7-O-glucuronide-5-O-rhamnoside, Quercetin-3-O-sambubioside*, Isosalipurposide (Phlorizin Chalcone), Hesperetin-7-O-glucoside*, Quercetin-3-O-rhamnoside(Quercitrin), Dihydromyricetin (Ampelopsin), 3,5,7-Trihydroxyflavanone (Pinobanksin), 3-O-Acetylpinobanksin, Apigenin-6-C-glucoside (Isovitexin), Naringenin chalcone; 2’,4,4’,6’-Tetrahydroxychalcone, Butin; 7,3’,4’-Trihydroxyflavanone, Cyanidin-3-O-glucoside (Kuromanin), Naringenin (5,7,4’-Trihydroxyflavanone), L-Glutamine, and L-Lysine were up-regulated (**Figures 9**, **10**). In addition, we identified distinct metabolites in amino acids and their derivatives, polyphenols, and fatty acids in berry, pulp, and seed. Using Venn diagrams to compare each group, we found only 41 metabolites were common among Q-R vs Q-RP, Q-R vs Q-RS, and Q-RP vs Q-RS, while only 20 metabolites were shared among Y-R vs Y-RP, Y-R vs Y-RS, and Y-RP vs Y-RS (**Figures 7B, C**). These findings suggest that the metabolites responsible for the variations between berry, pulp, and seed were significantly distinct. (**Supplementary Table S3**).

### 3.5 Antioxidant activity analysis of the raspberry from two plateaus

The most frequently employed techniques to evaluate the antioxidant capacity of foods include FRAP (ferric reducing activity of plasma), ABTS (2,2-azinobis(3-ethylbenzthiazoline-6-sulfonic acid)), DPPH (1,1-diphenyl-2-picrylhydrazyl), and ORAC (oxygen radical absorbance capacity). Furthermore, they carved two groups: a hydrogen atom transfer-based assay and an electron transfer-based assay (Burton-Freeman et al., 2016). Compared to common fruits like grapes, apples, and citrus, raspberries exhibit higher levels of antioxidant activity (Fu et al., 2011). As shown in **Table 1**, the antioxidant capacity of red raspberries in different plateaus was significantly different. For the DPPH scavenging performance test and FRAP total antioxidant test, Q-RS and Q-R showed higher antioxidant capacity than Y-RS and Y-R. For the ABTS radical scavenging test, Q-R and Q-RP showed higher antioxidant capacity than Y-R and Y-RP, which exhibited a higher ABTS radical scavenging capacity for Q-R (49.57 ± 1.26 μM TE/g DW), which was 10-fold higher than Y-R (4.68 ± 0.45 μM TE/g DW). The ORAC values of Q-RP and Q-RS were higher than those of Y-RP and Y-RS. In addition, the average ORAC values raised in the direction: seed < pulp < berry, the rise of DPPH, ABTS, and FRAP values were berry < pulp < seed, and the DPPH average values of berry, pulp, and seed were above 10-fold compared to ABTS values. These results may be due to the different reaction mechanisms in ABTS, DPPH, FRAP, and ORAC assays. These results indicated that there were significant differences in antioxidant activity between the same parts in red raspberries from different plateaus, with berry, pulp, and seeds of Qinghai red raspberries having higher antioxidant capacity compared to Yunnan red raspberries. In addition, the antioxidant capacity of the seed of Qinghai raspberry was the maximum (420.31 µM TE/g DW, respectively) of all, over 20 times higher than the least value (Yunnan berry). The order of the antioxidant capacity was seed > pulp > berry.

**Table 1:** Analysis of the antioxidant properties of Yunnan and Qinghai red raspberries.

Index	Y-R	Y-RP	Y-RS	Q-R	Q-RP	Q-RS
DPPH value (μg TE/g FW)	191.31 ± 7.57^d^	5345.70 ± 76.03^c^	5072.01 ± 50.23^b^	211.91 ± 11.77^d^	5022.00 ± 23.50^a^	5218.34 ± 78.16^c^
FRAP value (μΜ TE/g DW)	25.34 ± 7.10^e^	121.94 ± 6.01^c^	402.86 ± 16.11^b^	27.06 ± 5.94^e^	100.95 ± 5.08^d^	420.31 ± 8.65^a^
ORAC (%)	71.43 ± 1.07^a^	45.53 ± 0.04^d^	44.36 ± 0.06^e^	69.23 ± 1.11^b^	50.74 ± 0.06^c^	44.44 ± 0.03^de^
ABTS value (μΜ TE/g DW)	4.68 ± 0.45^f^	151.05 ± 1.71^d^	351.72 ± 5.71^a^	49.57 ± 1.26^e^	172.07 ± 5.99^c^	326.72 ± 10.09^b^

Different lowercase letters indicate significant differences at *p* < 0.05. FW, fresh weight; DW, dry weight. The same applies below.

### 3.6 Antioxidants and related metabolites correlation analysis

**Figure 11:**
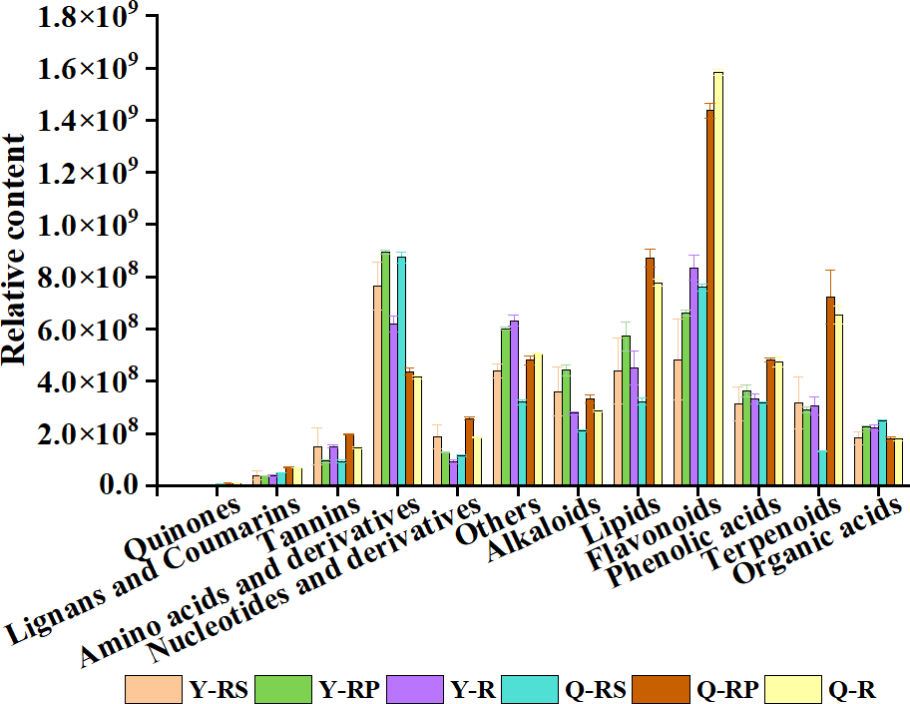
The relative content of each class of metabolites of the raspberry from different plateaus and parts. Comparison of the relative content of each category of metabolites in Y-RS, Y-RP, Y-R, Q-RS, Q-RP, and Q-R.

To reveal potential correlations between the metabolite profiles of red raspberries and their parts and antioxidant activities (**Table 1**), we compared the relative content of each class of metabolites. As shown in **Figure 11**, Y-R, Y-RP, Y-RS, Q-R, Q-RP, and Q-RS significantly differed in the relative contents of flavonoids, phenolic acids, amino acids and derivatives, lipids, and terpenoids. Unlike Y-R, Y-RP, and Y-RS, the accumulation of phenolic acids and flavonoids was higher in Q-R, Q-RP, and Q-RS. Additionally, in comparison to Y-RS, Q-RS has larger concentrations of flavonoids, amino acids and their derivatives.

### 3.7 Phenolic compounds composition in raspberry from two plateaus and their relation to antioxidant activity

In the current study, phenolic compound composition and concentration were evaluated in order to identify possible metabolites responsible for variations in antioxidant activity in raspberries and their parts from distinct plateaus.

#### 3.7.1 Flavonoids

A total of flavonoid metabolites were identified (28 flavanols, 13 flavanonols, 75 flavones, 28 anthocyanidins, 125 flavonols, 34 flavanones, 15 chalcones, 1 dihydroisoflavones, 7 isoflavones, 11 other flavonoids, of which 8 chalcones (4,4'-dihydroxy-2'-methoxychalcone, sieboldin, Phloretin-4'-O-glucoside (Trilobatin), Isosalipurposide (Phlorizin Chalcone), 3,4,2',4',6'-Pentahydroxychalcone-4'-O-glucoside, Phloretin-4'-O-(6''-Caffeoyl)glucoside, Phloretin-4'-O-(6''-p-Coumaroyl)glucoside, 2,4,2',4'-tetrahydroxy-3'-prenylchalcone), a dihydroflavone (Pinocembrin-7-O-(2''-O-arabinosyl)glucoside), (Diosmetin (5,7,3'-Trihydroxy-4'-methoxyflavone), Kaempferol-3-O-glucoside-7-O-rhamnoside*) were the most abundant flavonoids. Compared to the Y-R, Kaempferol-3-O-sambubioside and 6,7,8-tetrahydroxy-5-methoxyflavone were found to be specific to Q-R. Compared to the Q-R, 3,5,4'-Trihydroxy-7-methoxyflavone (Rhamnocitrin) was found to be specific to Y-R. Compared with Y-RP, there are flavonoids (Kaempferol-3-O-sambubioside, Kaempferol-3-O-(6''-Malonyl)glucoside-7-O-Glucoside) that are specific to Q-RP, 3,5,4’-trihydroxy-7-(rhamnocitrin) and 3,5,4'-Trihydroxy-7-methoxyflavone (Rhamnocitrin) that are specific to Y-RP. 3,5,4’-Trihydroxy-7-methoxyflavone (Rhamnocitrin) and Eriodictyol-7-O-glucoside* are specific to Y-RP, and 82 flavonoids are specific to Q-RS compared to Y-RS.

**Figure 12:**
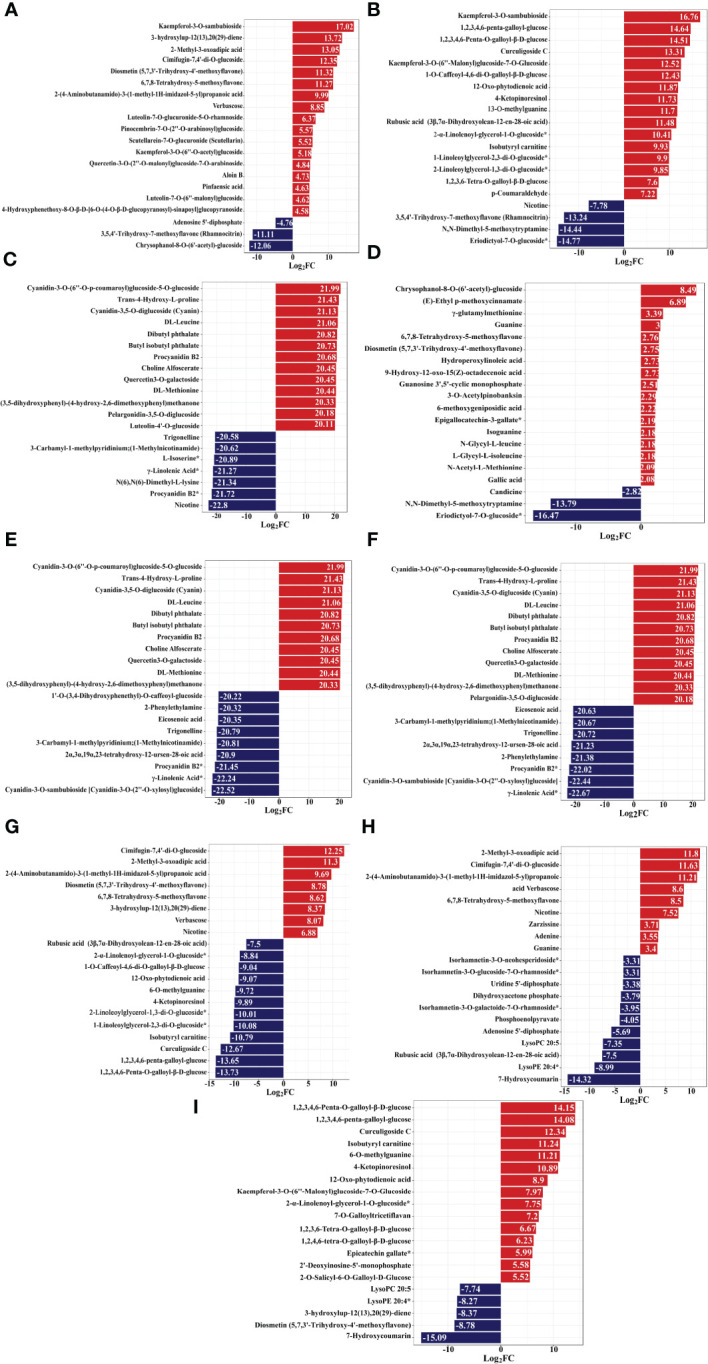
Variance multiplier bar chart, the horizontal coordinate is the value of the difference multiplier of the difference metabolite taken as logarithm with a base of 2. The vertical coordinate is the difference metabolite. Red represents up-regulation of metabolite content, blue represents down-regulation of metabolite content. Y-R vs Q-R, Y-RP vs Q-RP, Y-RS vs Q-RS, Q-R vs Q-RP, Q-R vs Q-RS, Q-RP vs Q-RS, Y-R vs Y-RP, Y-R vs Y-RS, and Y-RP vs Y-RS **(A–I)**.

Notably, the present study focused on the berry, pulp, and seeds of raspberries from two plateaus. Among them, compared to Y-R, flavonoids in core difference metabolites. For instance, Luteolin-7-O-glucuronide-5-O-rhamnoside, Quercetin-3-O-sambubioside*, Isosalipurposide (Phlorizin Chalcone), Quercetin-3-O-rhamnoside(Quercitrin), Dihydromyricetin (Ampelopsin), 3,5,7-Trihydroxyflavanone (Pinobanksin), 3-O-Acetylpinobanksin, Apigenin-6-C-glucoside (Isovitexin), Naringenin chalcone; 2’,4,4’,6’-Tetrahydroxychalcone, Butin; 7,3’,4’-Trihydroxyflavanone, Naringenin (5,7,4’-Trihydroxyflavanone) (log_2_FC = 6.37, 3.53, 3.31, 1.43, 1.77, 1.45, 4.32, 2.77, 1.52, 1.51, 1.46) were found at higher levels in Q-R (**Figure 12A**). Compared to Y-RP, the relative contents of Isosalipurposide (Phlorizin Chalcone), Hesperetin-7-O-glucoside*, Dihydromyricetin (Ampelopsin), 3,5,7-Trihydroxyflavanone (Pinobanksin), and Naringenin chalcone; 2’,4,4’,6’-Tetrahydroxychalcone (log_2_FC = 3.45, 2.22, 1.11, 1.49, 1.34) were significantly higher in Q-RP (**Figure 12B**). Compared to Q-RS, the Cyanidin-3-O-glucoside (Kuromanin) (log_2_FC = 6.22) contents were higher than that in Y-RS (**Figure 12C**). Based on these findings, it can be inferred that certain substances in the pathway of flavonoid biosynthesis may be influenced by environmental factors. As a result, the variations in the types and quantities of flavonoids present in the raspberries from the two plateaus could potentially result in varying levels of antioxidant activity.

#### 3.7.2 Phenolic acids

Phenolic acids possess antioxidant properties and are typically found in plant cell walls, where they are closely associated with polysaccharides (Río Segade et al., 2019). Phenolic acids in core difference metabolites, levels of chlorogenic acid (3-O-caffeoylquinic acid)* (log_2_FC = 2.78) and 5-O-p-coumaroylquinic acid* (log_2_FC = 1.85) were significantly higher in Q-RP than those in Y-RP. Therefore, the composition and amount of phenolic acids in raspberries from different regions may influence their antioxidant capacity. This could be due to variations in growing conditions, such as climate, soil type, and altitude, which can affect the plant’s ability to produce and accumulate phenolic compounds.

### 3.8 Amino acids and their derivatives compounds composition in raspberry from two plateaus and their relation to antioxidant activity

**Figure 13:**
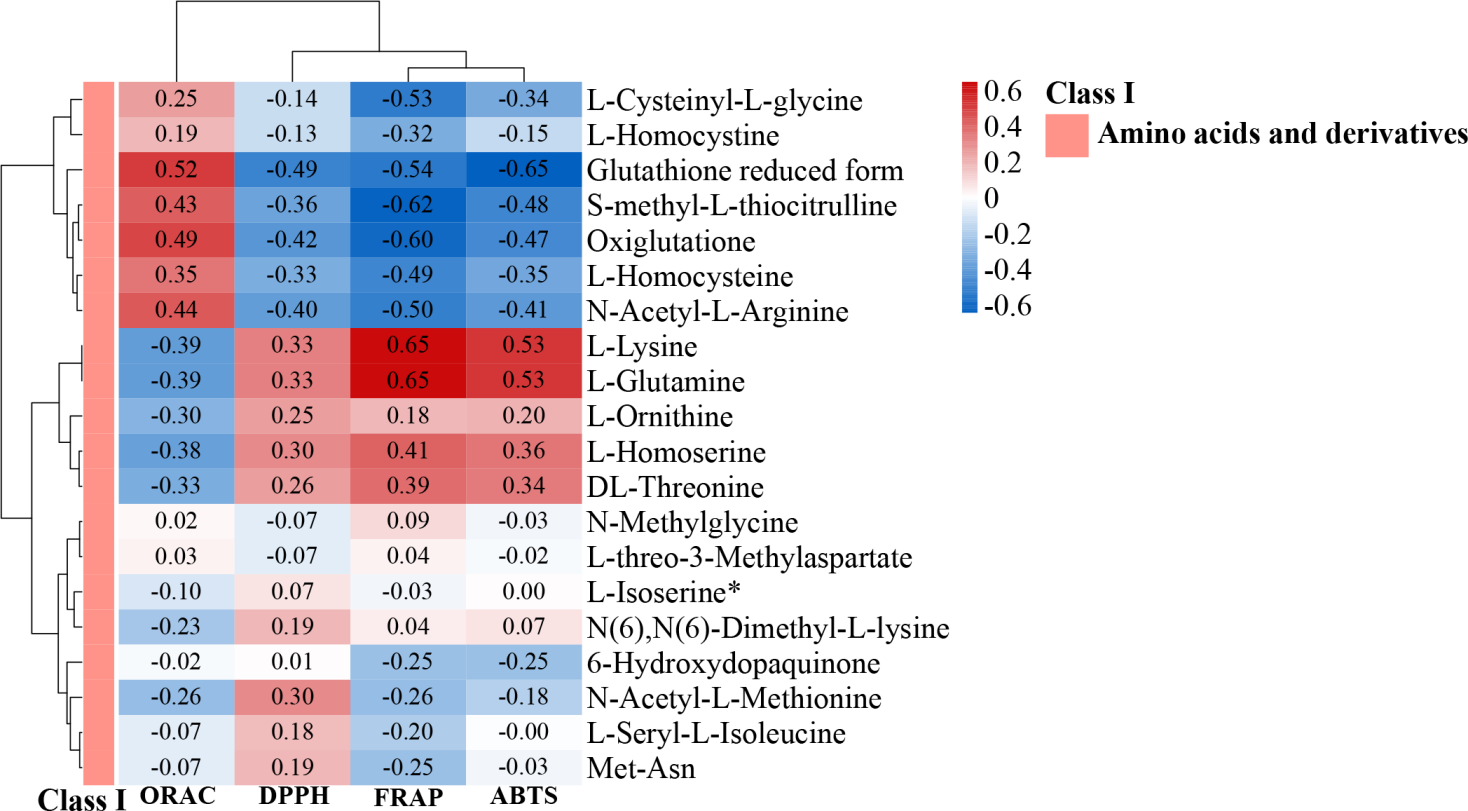
Heat map of the correlation between amino acids and their derivatives in core metabolites and antioxidants. Different letters above columns and the color of the column indicate the correlation, with the larger and the redder the color and the greater the correlation coefficient.

Plants are the only organisms capable of synthesizing the essential amino acids (leucine, isoleucine, methionine, phenylalanine, arginine, histidine, tryptophan, valine, threonine, and lysine) (Kumar et al., 2017). The amino acid and derivative compositions in Y-RS, Y-RP, Y-R, Q-RP, and Q-R detected were very comparable, only with Q-RS containing 24 unique amino acids and derivatives. Notably, among the top 20 differential up-regulated or down-regulated metabolites (Y-RS and Q-RS), 7 compounds were amino acids and their derivatives (**Figure 12C**). In Qinghai berries, L-Lysine and L-Glutamine were mainly present in the seeds, and the more antioxidant properties of the seeds might be related to the amino acid content. To gain a better understanding of the antioxidative metabolite composition, antioxidant activity was measured. Therefore, Spearman’s rank correlation tests were used to test for correlation analysis of the amino acids and their derivatives DAMs with ORAC, DPPH, FRAP, and ABTS. We can find from **Figure 13** that different amino acids and their derivatives have different correlations with antioxidant capacity, with L-Lysine and L-Glutamine showing the most significant correlation with antioxidant properties. DPPH, FRAP, and ABTS were clustered, indicating that these three antioxidant assays had positive correlations and showed the lowest correlation with ORAC assay.

### 3.9 Metabolite-metabolite correlation

**Figure 14:**
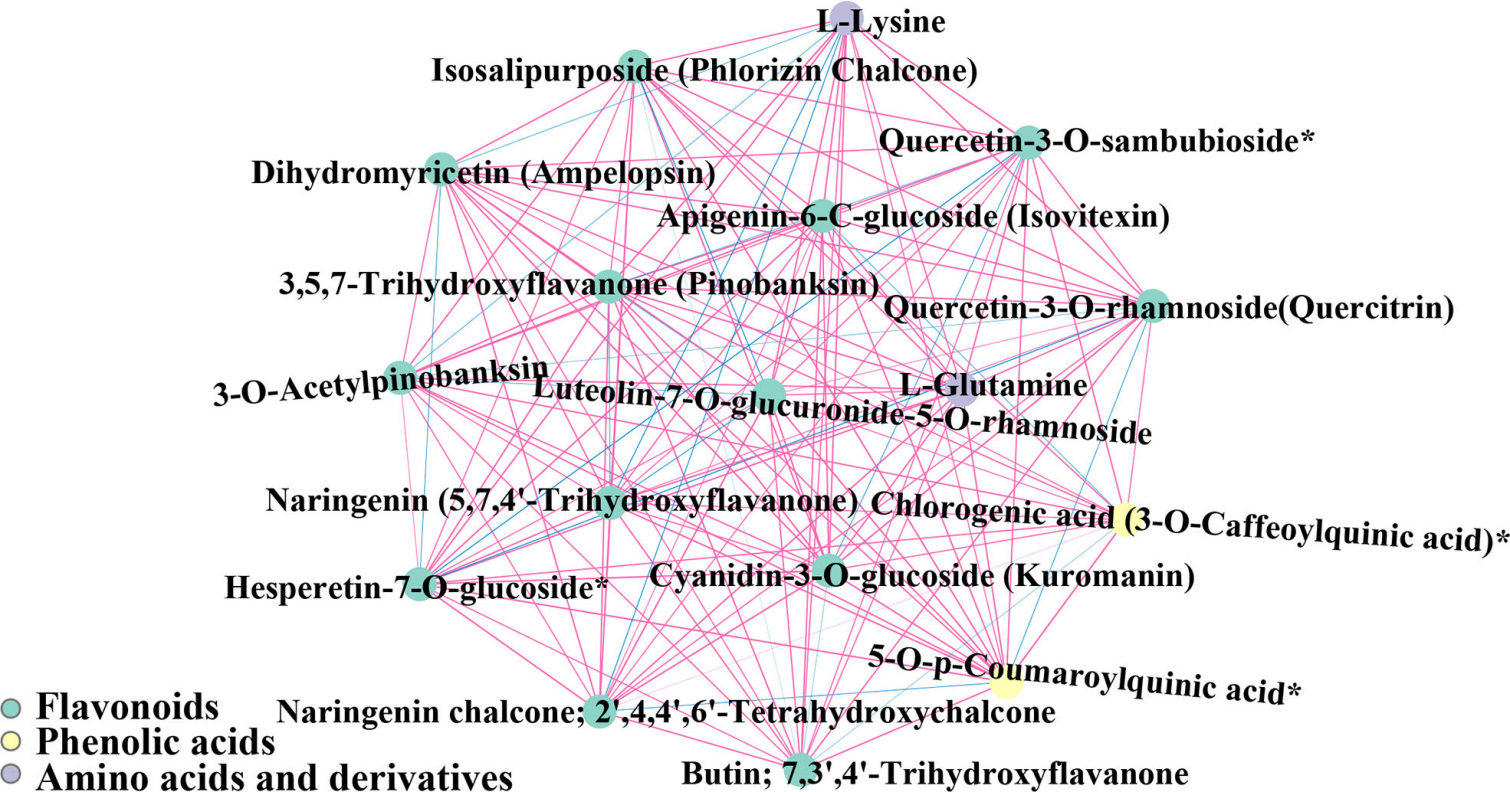
Map of significant seed metabolite-metabolite correlations. Metabolites are represented by circles, and the same color indicates metabolites in the same metabolic function group. Correlations are indicated by connected lines. Positive correlations are red and negative correlations are blue. The thickness of the line represents the magnitude of the absolute value of Pearson’s correlation coefficient r. The thicker the line, the larger the |r|.

289 pairwise correlation values were obtained for 17 different metabolites in red raspberry from two plateaus, of which flavonoids and phenolic acids were determined to be significant (r > 0.80, *p* < 0.05) (**Figure 14**). Furthermore, two amino acids (L-Glutamine and L-Lysine) and Cyanidin-3-O-glucoside (Kuromanin) were found to be significantly correlated. In antioxidant assays, they demonstrated synergism. The presence of amino acid metabolites in significant metabolite-metabolite correlations suggested that amino acids positively effect the antioxidant activity.

## 4 Discussion

Because of their highly functional ingredients content and antioxidant activities, raspberries are becoming increasingly popular. However, differences exist in the metabolites of raspberries grown in different environments. Therefore, the present study was designed to provide a widely targeted metabolomic analysis of the relationship between metabolites and antioxidants in raspberries and parts.

### 4.1 Differential metabolites between raspberries from two plateaus

Flavonoids, phenolic acids, amino acids and derivatives, and lipids were the most significant differential metabolites between the two plateaus. About half of the 264 key significantly differential metabolites were flavonoids and phenolic acids. The analysis of metabolic pathways for the different metabolites in Y-R vs Q-R, Y-RP vs Q-RP, and Y-RS vs Q-RS groups showed a significant involvement of flavonoid biosynthesis, anthocyanin biosynthesis, biosynthesis of amino acids, and flavone and flavonol biosynthesis metabolic pathways. This suggests that there are notable differences between the two plateaus in terms of weather, rainfall, altitude, and temperature. Previous studies found during red raspberry development, in stage 2 with stage 3 (25 DAFB) d after full bloom (DAFB), which mainly involves KEGG pathways related to flavonoid biosynthesis and phenylpropanoid biosynthesis (Huang et al., 2022). This previous study is consistent with the main pathway in the present study and suggests that antioxidant-related substances in raspberries are produced during the second stage of raspberry growth. Interestingly, linoleic acid metabolism was significantly up-regulated (*p* < 0.05) in the comparison of in Y-RP vs Q-RP group, indicating that the *γ*-Linolenic Acid* is the main differential metabolite. The study showed that the two plateaus growing environment causes drastic changes in polyphenols metabolites. Recent research by Grand View Research, Inc. has suggested that the worldwide polyphenols market is arriving at USD 2.9 billion by 2030 (Sarv et al., 2021). Polyphenols, as antioxidants from many berries and particularly raspberries. The mechanism of their antioxidant activity can be characterized by the direct scavenging or quenching of oxygen radicals or excited oxygen species and the inhibition of oxidative enzymes that produce these reactive oxygen species (Baby et al., 2018). As food supplements can enhance our body’s antioxidant defense system, reduce life-threatening diseases caused by oxidative stress, and greatly reduce the risk of cancer (Alarcón-Flores et al., 2013; Yang et al., 2018). The main determinants of the total antioxidant capacity of fruits such as berries are particularly rich in flavonoids (Panche et al., 2016). The flavonoid synthesis pathway involves the condensation of phenylpropanoid derivatives with malonyl-CoA, as reported by Ono et al. (2006). In addition, transcription factors play a role in regulating certain aspects of this pathway. According to Mudge et al. (2016), berries are known to have elevated concentrations of Quercetin-4’-O-glucoside, Quercetin-3, 4’-O-diglucoside, and Quercetin-3-O-rutinoside. Our study supports these previous findings, as we found a substantial amount of Quercetin-4’-O-glucoside in raspberries.

In order to investigate the key metabolites related to antioxidant capacity in raspberries from two different plateaus, a total of 264 overlapping differential metabolites were identified. Out of these, three differential metabolites associated with antioxidant activity were selected by using the up-regulated metabolites between different groups (Y-R vs Q-R, Y-RP vs Q-RP, and Y-RS vs Q-RS) as a criterion for screening. We found the main up-regulated flavonoids in Qinghai raspberry’s berry, pulp, and seed included Quercetin-3-O-rhamnoside (Quercitrin), Cyanidin-3-O-glucoside (Kuromanin), Naringenin (5,7,4’-Trihydroxyflavanone) (**Figures 10C, G**, and M). According to the research, Cyanidin-3-O-glucoside is responsible for the antioxidant properties of blackberries (Huang et al., 2022). Quercetin-3-O-rhamnoside could be a major contributor to antioxidant activity (Nie et al., 2020). Naringenin (5,7,4'-trihydroxyflavanone) is a naturally occurring bioactive flavanone, which is documented to have bioactive effects on human health, such as antidiabetic, immunomodulatory, anticancer, anti-inflammatory (Stec et al., 2020). These molecules responded particularly to increased antioxidant activity and, hence, can serve as indicators for selecting Qinghai berries.

The molecules known as α-amino acids consist of an α-carbon atom attached to an amino group (NH_2_), a carboxyl group (COOH), a hydrogen atom (H), and a side chain (R), where the NH2 group is connected to the α-carbon. Due to structural differences in the side chains, these amino acids’ antioxidation mechanisms and capacities vary (Xu et al., 2017). Other studies have reported that lysine with strong antioxidative capacity in 20 amino acids. Researchers found that tolerant citrus germplasm possesses a large number of amino acids with high antioxidant potential, such as lysine, tyrosine, phenylalanine, tryptophan, and asparagine (Killiny and Hijaz, 2016). The reported case illustrates that amino acids are associated with plant resistance to several abiotic and biotic stresses. In this study, one of the reasons for the higher antioxidant capacity of Qinghai raspberry may be due to the synthesis of a large number of amino acids with antioxidant capacity under biotic stress. Y-RS compared to Q-RS, we found the highest relative content of L-Lysine and L-Glutamine among amino acids and their derivatives in Qinghai raspberry seeds. L-Lysine is known to have a positive impact on antioxidant status by primarily boosting the effectiveness of the GSH and peroxidase systems, which are crucial components of the body’s antioxidant defense mechanism. By doing so, L-Lysine acts to eliminate free radicals and guard against the harmful effects of oxidative damage caused by these radicals (Al-Malki, 2015). In addition, oxidative stress and neuronal apoptosis were decreased by L-Glutamine (Luo et al., 2019), and It is a precursor for the generation of a variety of secondary defensive metabolites. Finally, amino acid metabolic pathways stimulated plant defense to increase resistance. Transgenic rice overexpressing *M. oryzae* Systemic Defense Trigger 1 showed this pattern. (Duan et al., 2021).

The correlation between amino acids and their derivatives in the core metabolites and antioxidants was analyzed, and L-Lysine and L-Glutamine showed a significant positive correlation with antioxidants. The strongest correlations were found between DPPH, ABTS, and FRAP assays, especially ABTS and FRAP. Then, ORAC had the lowest correlations. Unlike the others, the ORAC assay determined the kinetic action of antioxidants, which might explain the discrepancy. The DPPH, ABTS, FRAP, and ORAC assays gave comparable results for the antioxidant activity tested in extracts from guava fruit. The highest correlations were found between DPPH, ABTS, and FRAP assays, especially between ABTS and FRAP assays, a result previously reported by Thaipong et al. (2006). Another study by Dudonné et al. (2009) that evaluated the antioxidant properties of 30 industrial plant extracts using DPPH, ABTS, FRAP, SOD, and ORAC assays also obtained consistent results.

Correlation analysis of different metabolites is helpful to find the relationship between metabolites and discover potential key metabolic regulatory (Fukushima et al., 2011). The red raspberry metabolic network displayed a highly concerted interplay of amino acids, providing evidence for the essential conserved roles of amino acids in plant’s metabolism. These are highly positive correlations because the correlated metabolites were either participating in the same metabolic biosynthesis of amino acids reaction as a substrate-product or were controlled by the same regulatory enzymes’ activities. Also, L-Lysine and L-Glutamine were found to be highly positively correlated in our study. The information on these significant metabolite correlations could help better understand red raspberry’s metabolic regulatory network and potentially discover novel metabolic pathways.

Furthermore, the synergistic effect among natural antioxidants is one of the mechanisms by which amino acids exert their antioxidant activity, also known as the synergistic antioxidant effect with other antioxidants (Zhang et al., 2022). Due to the increase in the content of amino acids and phenolic compounds and their derivatives. Their derivative content induces the activity of enzymes related to amino acids and phenolic compounds, and the expression of genes related to amino acid and phenolic metabolism contributes to the improvement of antioxidant capacity (Wang et al., 2021). The above research indicated that amino acids and polyphenols have synergistic effects in terms of antioxidant activity. In our correlation analysis, L-Lysine, L-Glutamine, and Cyanidin-3-O-glucoside (Kuromanin) were found to have strong correlations demonstrating their potential to be used as a potential to increase antioxidant capacity. Taken together, besides some specific flavonoids and phenolic acids, the reason for the strong antioxidant capacity of raspberry seeds and Qinghai raspberry may be related to the fact that L-Lysine and L-Glutamine content and synergistic effect with polyphenols.

Plant antioxidant defense mechanism has attracted many researchers’ attention (Mazur et al., 2014; Bhattacharjee, 2019). Some metabolites as metabolic antioxidant defense systems are key components of plant growth during adaptation to biotic and abiotic stress conditions, such as fatty acids, amino acids, phenolic acids, flavonoids, and anthocyanins (colored pigments) (Wink, 2008). For most plants, external factors (light, temperature, precipitation) can significantly affect their ability to synthesize secondary metabolites (Yang et al., 2018). Other studies have found that cultivar, climate, and growing conditions all have an impact on the quality and chemical composition of ten red raspberry genotypes (Mazur et al., 2014). Yang et al. (2020) also found that the most of the berries harvested from the dried temperate continental climate plateaus present higher antioxidant activity than the ones harvested from the continental climate (Georgescu et al., 2022). Recent research on blueberry and chokeberry revealed that the metabolite composition also depends on geo-climatic conditions, especially latitude. Berries harvested in geo-climatic zones of different latitudes have relatively high amino acid contents (Sim et al., 2017). In many cases, UV irradiation induces the processing of secondary metabolites, such as UV irradiation significantly increases flavonoid content (Chaves et al., 1997; Umek et al., 1999; Afshar et al., 2022), enhancing the total accumulation of anthocyanins (Gläßgen et al., 1998; Araguirang and Richter, 2022). This phenomenon, known as the defensive effect, is attributed to the ability of ROS scavenging as well as defending plants from excessive sunlight to growth enhancement (Dehghanian et al., 2022). These studies suggest that the environment induces the metabolites of raspberries grown in different environments. Climatic conditions are a major influencing factor. In our study, we found that the antioxidant properties of Qinghai raspberries were higher than those of Yunnan raspberries, which may be due to Qinghai being located in QZP with low temperature, high altitude, and long hours of sunlight of climatic conditions. These lead to increased production of ROS in plant cells to achieve antioxidant defense and increased content of metabolites with antioxidant properties. Primarily, flavonoid, phenolic acids, and amino acids and their derivatives were responses to abiotic stresses such as ultraviolet radiation, drought resistant, and cold resistance. Considering that Qinghai raspberry has strong antioxidant properties and the seeds are rich in secondary metabolites, the transcriptome and metabolite profile can be jointly analyzed to find how related genes regulate metabolites to have different expressions in Qinghai raspberry, thus promoting the diversity and variability of nutrients and bioactive compounds in Qinghai raspberry.

### 4.2 Differential metabolites among raspberries in different parts

The current study found that working with Qinghai red raspberry and Yunnan raspberry, the major part of the weight of fresh raspberries included pulp, therefore, it is expected that the berry and pulp could have comparable composition. In recent years, researchers investigated the correlation analysis and observed positive correlations between the antioxidant assays and polyphenolic groups of raspberries (Basu and Maier, 2016; Carmichael et al., 2021). In this study, the antioxidant activity was found in berry and pulp samples and may be related to the high concentration of 4 anthocyanidins (Pelargonidin-3-O-glucoside, Peonidin-3-O-glucoside, Cyanidin-3-O-sambubioside [Cyanidin-3-O-(2’’-O-xylosyl) glucoside], Cyanidin-3-O-(6’’-O-p-Coumaroyl) glucoside).

The KEGG pathway enrichment analysis revealed that the differential metabolites between Q-R and Q-RP were mostly involved in phenylpropanoid biosynthesis, linoleic acid metabolism, biosynthesis of various plant secondary metabolites, and purine metabolism. By clustering all the differential metabolites in these pathways, in the Q-RP, phenolic acids (p-Coumaryl alcohol, Coniferaldehyde, p-Coumaraldehyde, Coniferyl alcohol), and amino acids and derivatives (L-Phenylalanine) are overrepresented in the phenylpropanoid biosynthesiss (**Supplementary Figure S3A**). Such as lignans and Coumarins (3,4-Dihydrocoumarin, Pinoresinol), phenolic acids (Gallic acid, Coniferyl alcohol) and amino acids and derivatives (L-Phenylalanine, L-Methionine) were clustered in the biosynthesis of various plant secondary metabolites as well as their derivatives were detected at a high level (**Supplementary Figure S3B**). Free fatty acids (13-KODE; (9Z,11E)-13-Oxooctadeca-9,11-dienoic acid, 12,13-Epoxy-9-Octadecenoic Acid, 7S,8S-DiHODE; (9Z,12Z)-(7S,8S)-Dihydroxyoctadeca-9,12-dienoic acid, 9(10)-EpOME;(9R,10S)-(12Z)-9,10-Epoxyoctadecenoic acid) which were highly detected in the Q-RP might attribute the antioxidant activity. In this study, linoleic acid metabolism and phenylpropanoid biosynthesis were significantly enriched in Y-R and Y-RP (*P* < 0.05). Caffeic acid, cinnamic acid, ferulic acid, and sinapic acid were up-regulated in Y-RP (**Supplementary Figure S3D**). 12 free fatty acids were significantly up-regulated in Y-R (**Supplementary Figure S3C, E**). This also showed that the antioxidant activity in berry and pulp causes differences in flavonoid and unsaturated fatty acids metabolites.

Compared to the berry and pulp, seeds hold higher concentrations of flavones (Hispidulin-7-O-glucoside (Homoplantaginin)), free fatty acids (9(10)-EpOME;(9R,10S)-(12Z)-9,10-Epoxyoctadecenoic acid, 9,10,13-Trihydroxy-11-Octadecenoic Acid), phenolic acids (Raspberry ketone glucoside, Ethyl ferulate, 4’-Hydroxypropiophenone, Vanillin acetate), flavanones (hesperetin-7-O-rutinoside (hesperidin)*, hesperetin-7-O-neohesperidoside(Neohesperidin)*), tannin (pterocaryaninB), amino acids and derivatives (L-Lysine, L-Glutamine), making the seed fraction a considerable source of natural antioxidants. These findings prove that the seed part obtained from raspberry can supply a valuable source of functional ingredients with antioxidant properties for functional food and pharmaceutical purposes. Polyunsaturated fatty acids (PUFAs), including *γ*-Linolenic Acid* play multiple key roles in host defense and immunity, including anti-inflammation and antioxidative activity (Michalak et al., 2016). In addition, fatty acids in Q-RS mainly include Elaidic Acid, LysoPC 18:3*, LysoPC 18:3(2n isomer)*, *α*-Linolenic Acid*, and LysoPC 18:1*. In another study, Caidan et al. (2013) also reported different profiles consisting of palmitic, linolenic, linoleic, and stearic acids. Compared with the seeds, berry, and pulp of Qinghai raspberry, 325 metabolites are unique to Q-RS, including 67 phenolic acids, 52 terpenoids, 14 lipids (8 free fatty acids), 24 amino acids and their derivatives, 82 flavonoids, and 4 tannins (**Supplementary Table S4**). More than half of the total metabolites are relatively higher in the pulp. Therefore, the higher antioxidant capacity of Qinghai raspberry seeds is inseparable from their unique metabolites. As shown in **Figure 12**, the results of the top 20 metabolites in terms of the fold of difference in each group comparison. As presented in **Figure 12E and F**, Comparison of Q-RP and Q-RS, Q-R and Q-RS, Cyanidin-3-O-(6’’-O-p-coumaroyl) glucoside-5-O-glucoside, Cyanidin-3,5-O-diglucoside (Cyanin), Quercetin3-O-galactoside (log_2_FC = 21.99, 21.13, 20.45) are specific to Q-R and Q-RP. Tannins, as metabolic antioxidants, allows plants to respond to adverse environmental conditions (Casacchia et al., 2019). Procyanidin B2 and Butyl isobutyl phthalate (log_2_FC = 20.68, 20.73) were only detected in Qinghai raspberries’ seed. Besides, Procyanidin B2 is described as an important and unique anthocyanin in the red raspberry seed. Notably, in the present study, among the top 20 differential up-regulated or down-regulated metabolites (Q-RP and Q-RS, Q-R and Q-RS). Trans-4-Hydroxy-L-proline, DL-Leucine, and DL-Methionine (log_2_FC = 21.43, 21.06, 20.44) were unique amino acids and their derivatives to seeds in Qinghai raspberries. The difference in composition and content of flavonoids, phenolic acid, amino acids and their derivatives, and fatty acids might lead to strong antioxidant activities in the seeds from Qinghai raspberry.

The FAO reported that the world population is projected to grow by 34%, from 6.8 billion today to 9.1 billion in 2050. The total production of fruits (650,684 tons) was adequate for the year 2017 (Mishra et al., 2022). However, it is not easy to evaluate the availability and cost of fruits on account of these variable population estimates. Therefore, greater production will be needed, particularly raspberries with a high antioxidant capacity, by the years 2025 and 2050, as the consumer trust that an appropriate diet reduces illness with decreased costs of pharmaceuticals is also considered to promote the demand for raspberries. Atkinson et al. (2013) proved that an inadequate winter chill induced the declining yield of perennial fruit species in Europe and the Americas. Moreover, the surveys of the FAO in 2017 have reported that raspberry productivity was 2.19 tons per ha in Serbia (Europe, an annual average temperature: 5-15°C), while this was only 0.64 tons per ha in Mexico (Americas, an annual average temperature: 16-28°C). Therefore, although Qinghai is located in Qinghai-Xizang plateau, its climate and geographical conditions are appropriate for planting raspberries, and the quality of raspberry grown in this area are better than in Yunnan. In addition, to meet the demand for functional substances and prevent human diseases in the context of global population growth. Therefore, we suggest that Qinghai raspberries with high antioxidant substances contents are widely grown in Qinghai. As a significant part of phytochemicals remain in the raspberry seed fraction, particularly Qinghai raspberry, it can be a potential source of functional ingredients to increase the utilization of raspberry.

## 5 Conclusions

In this study, LC-MS-based metabolomics and biochemical indicators were used to investigate the antioxidant properties of raspberries and their parts from two different plateaus. The results showed that there were metabolic differences between Qinghai raspberry and Yunnan raspberry, with different pathways being affected in each. The antioxidant capacity of the raspberry was found to be primarily related to the content and types of flavonoids, free fatty acids, and phenolic acids in the berry and pulp. In addition, the unique composition and content of flavonoids [Cyanidin-3-O-(6’’-O-p-coumaroyl) glucoside-5-O-glucoside, Cyanidin-3,5-O-diglucoside (Cyanin), and Quercetin3-O-galactoside], tannin (Procyanidin B2), phenolic acids (Butyl isobutyl phthalate), amino acids (L-Lysine and L-Glutamine) and fatty acids found in the seeds of Qinghai raspberry were shown to contribute to their strong antioxidant activities. The study suggests that the red raspberry seeds fraction could be a valuable source of functional ingredients for increasing the utilization of red raspberry. Overall, the research provides new insights into the chemical composition of red raspberry and its parts, and highlights the potential health benefits associated with its consumption.

## Data availability statement

The original contributions presented in the study are included in the article/**Supplementary Material.** Further inquiries can be directed to the corresponding authors.

## Author contributions

XR conceived the project, data analysis, and edited the manuscript. YS designed the research, reviewed and edited the manuscript, made strict revisions to the grammar of the manuscript and performed the funding acquisition. SW, JW, and DX revised the paper. YY performed the research and discussed the results. All authors contributed to the article and approved the submitted version. 
