# Supplementary material for: Widely targeted metabolome profiling of different plateau raspberries and berry parts provides innovative insight into their antioxidant activities
Source: Front Plant Sci. 2023 Mar 13;14:1143439. doi: 10.3389/fpls.2023.1143439 (PMC10042140; doi:10.3389/fpls.2023.1143439)
Supplement: Supplementary file 1 [file DataSheet_1.pdf]

Supplementary Figure

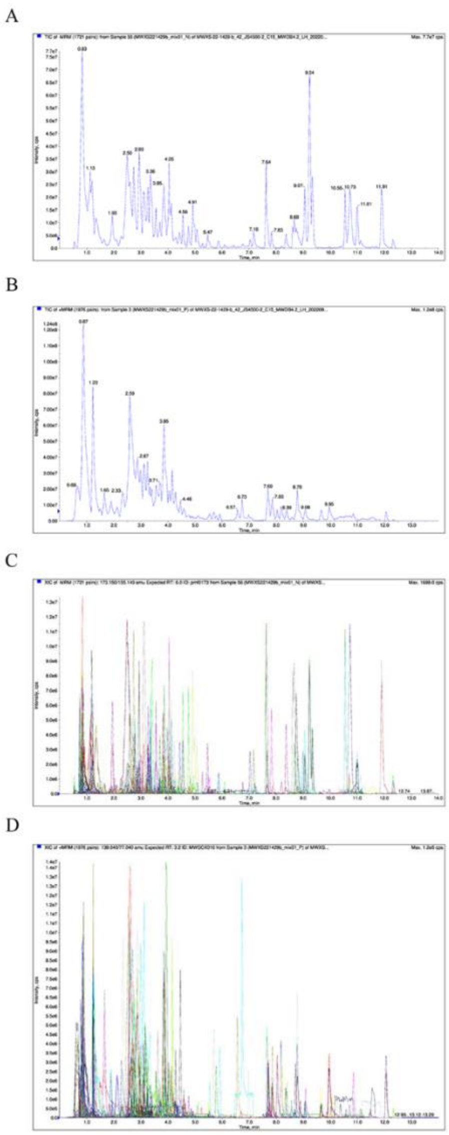

Supplementary Figure S1. Total ion current of one quality control sample by mass spectrometry detection (A, B) and multi-peak detection plot of metabolites in the multiple reaction monitoring mode (C, D). (A) and (C) were acquired in negative ionization mode. (B) and (D) were acquired in positive ionization mode.

A

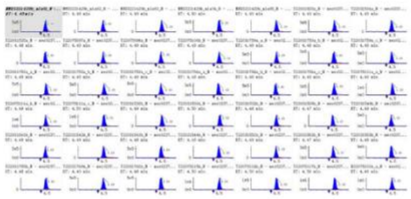

B

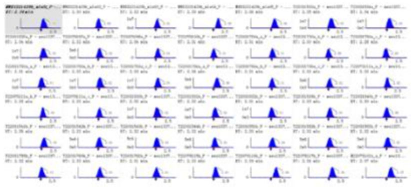

Supplementary Figure S2. Integral calibration chart for quantitative metabolite analysis. A was the detection in negative ion mode, B was acquired in positive ionization mode.

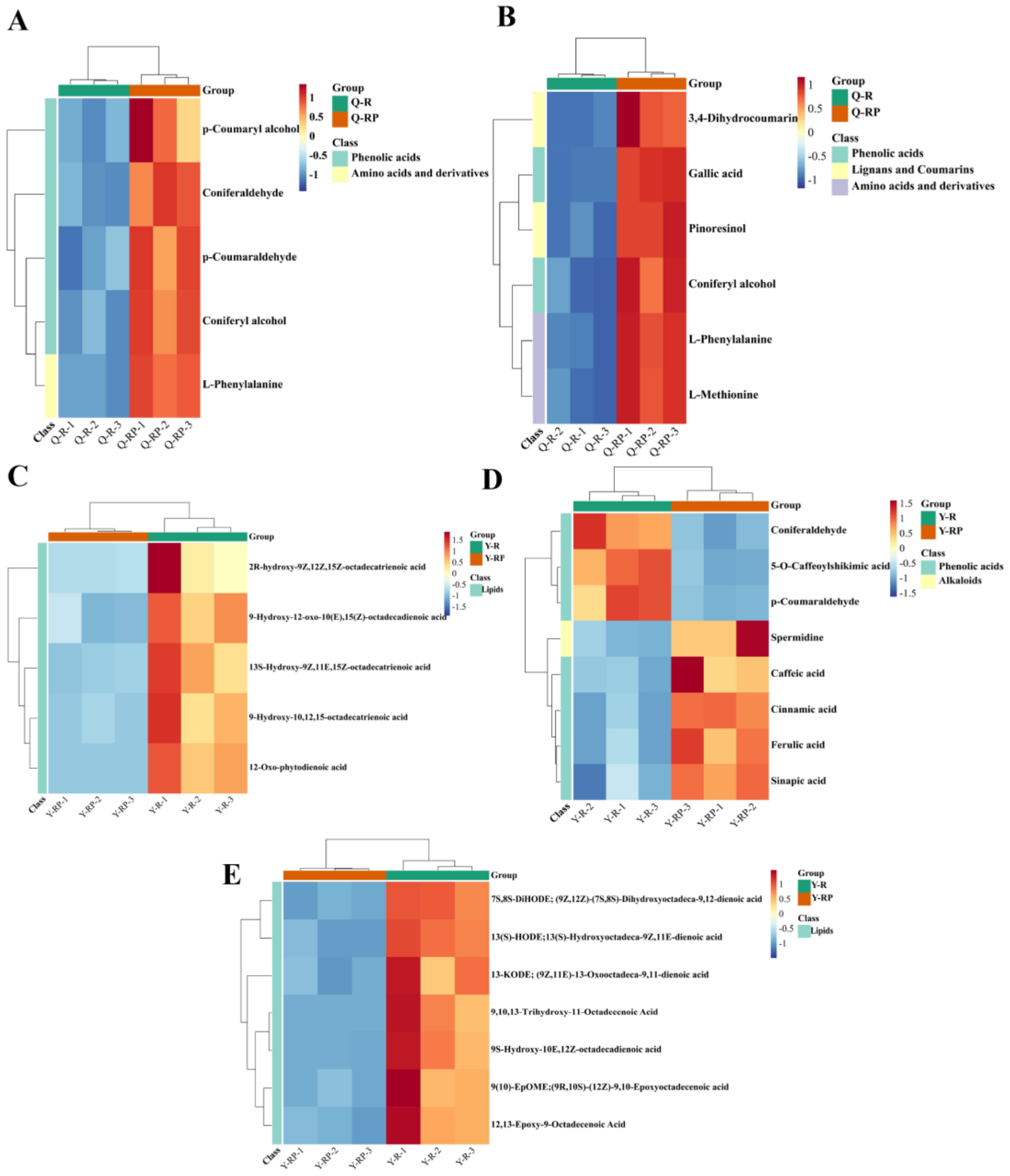

Supplementary Figure S3. Heat map of differential metabolite clustering in the KEGG pathway. Metabolites that involved in phenylpropanoid biosynthesis, (A); biosynthesis of various plant secondary metabolites, (B); in Q-R and Q-RP. Metabolites that involved in, alpha-Linolenic acid metabolism, (C); phenylpropanoid biosynthesis, (D); linoleic acid metabolism, (E); in Y-R and Y-RP.
